# An efficient fusion algorithm combining feature extraction and variational optimization for CT and MR images

**DOI:** 10.1002/acm2.12882

**Published:** 2020-04-19

**Authors:** Qinxia Wang, Xiaoping Yang

**Affiliations:** ^1^ School of Science Nanjing University of Science & Technology Nanjing P.R.China; ^2^ Department of Mathematics Nanjing University Nanjing P.R.China

**Keywords:** image fusion, primal‐dual algorithm, saliency detection, structure tensor, variational model

## Abstract

In medical image processing, image fusion is the process of combining complementary information from different or multimodality images to obtain an informative fused image in order to improve clinical diagnostic accuracy. In this paper, we propose a two‐stage fusion framework for computed tomography (CT) and magnetic resonance (MR) images. First, the intensity and geometric structure features in both CT and MR images are extracted by the saliency detection method and structure tensor, respectively, and an initial fused image is obtained. Then, the initial fused image is optimized by a variational model which contains a fidelity term and a regularization term. The fidelity term is to retain the intensity of the initial fused image, and the regularization term is to constrain the gradient information of the fused image to approximate the MR image. The primal‐dual algorithm is proposed to solve the variational problem. The proposed method is applied on five pairs of clinical medical CT and MR‐T1\MR‐T2 images, and the comparison metrics SF, MI, $Q^{AB/F}$ , $Q_{W}$ , and VIFF are calculated for assessment. Compared with seven state‐of‐the‐art methods, the proposed method shows a comprehensive advantage in preserving the salient intensity features, as well as texture structure information, not only in visual effects but also in objective assessments.

## 1 INTRODUCTION

The rapid development of computer technology promotes the progress of medical imaging, clinic practices, and medical science. Different imaging methods are complementary and provide more information than any individual among those. For example, computed tomography (CT) can capture the high density bone structures, while magnetic resonance imaging (MRI) can provide clear soft tissue structures in organs, such as brain, heart, and liver. Medical image fusion is the process of combining various information from multimodality medical images to obtain a fused image in order to increase clinical applicability for medical diagnosis and treatment of medical problems. Medical image fusion methods have been applied in human organs, including brain, breast, liver, and other organs.^1^, ^2^

Researchers have paid more attention to fuse multimodal medical images in order to obtain a single image with more information for physicians. Medical image fusion can be performed through the multiscale transform (MST) methods.^3^, ^4^ The laplace pyramids (LP)^5^, ^6^ and wavelet transforms (WT)^7^, ^8^, ^9^ are two kinds of classical MST methods. The multiscale geometric analysis (MGA) tools have been employed in image fusion, including curvelet transform,^10^ non‐subsampled contourlet transform (NSCT),^11^, ^12^ and non‐subsampled shearlet transform (NSST).^13^, ^14^ Moreover, the MST methods can extract difference in resolution information, which can be used to combine with other image processing methods, such as sparse representation (SR)^15^ and pulse coupled neural network (PCNN).^16^

Two‐scale image fusion methods can decompose each input image into a base layer and a detail layer through a filter method. The guided filter‐based fusion method (GFF)^17^ first extracts the average contrast to build the weight maps, then the guided filter is used to refine the weight maps of the two layers, and the final fused image is generated by the weighted average method. In Ref. [18], the authors established a gradient domain guided filter‐based fusion model, and the weight maps are obtained by combing multiple visual features including contrast, sharpness, and structure saliency measures.

Medical image fusion can also be regarded as optimization problems. The variational image fusion methods always contain two terms. The first term is a fidelity norm used to constrain the gray approximation between the fused image and input images, and the second term is a regularization constraint. In Ref. [19, 20, 21], the authors utilized the TV norm as the regularization term, while the fidelity term is chosen to be the quadratic norm or $L_{1}$ norm. As a higher‐order regularization constraint, the total generalized variation (TGV) is applied for image fusion as it can suppress the staircasing effect.^22^, ^23^

As two of most commonly used medical imaging modalities, CT and MR images are frequently combined for medical image analysis. CT images are density imaging, and can capture bone structures with high intensity. MR images can capture clear soft tissue structure information, especially the tumors which have high intensity in MR‐T2 images. For CT and MR image fusion, the fusion strategies of most existing methods usually extract the same features from source images and use them to construct weight maps which may lead to the “feature average” problem. In order to extract different and complementary features from source CT and MR images, we propose a novel fusion method which can keep these extracted features enhancement in the fused images. For example, the proposed method can extract not only the high intensity features (such as bone structures and tumor) from CT and MR brain images, but also the soft tissue texture structures from MR images. As a result, we can obtain fused images which preserve the bone structures, tumor features, as well as the soft tissue texture information in CT and MR images.

In this study, a two‐stage fusion framework is proposed for CT and MR images. First, the contrast‐based method and structure tensor are used to extract salient intensity information and geometry structures of medical images, respectively. The extracted intensity and structure features are used to construct weight maps and obtain an initial fused image. Then, a variational model is proposed to optimize the initial fused image. The variational framework consists of two terms: the fidelity term which is used to retain the saliency intensity information, and the regularization term which is used to constrain the approximation of gradient information between the fused image and MR image, and keep the fused image smooth at the same time. In the numerical experiments, the proposed method is compared with seven state‐of‐the‐art medical fusion methods on several pairs of CT and MR images. The experimental results show that the proposed method can well preserve the clear bone structures, tumor features, as well as the soft tissue texture information from the source images, and get a good visual quality.

The rest of the paper is organized as follows. In Section 2, we introduce some related works about saliency detection and structure tensor. In Section 3, an initial fusion image is generated with the weighted fusion method, and then it is optimized by the proposed variational fusion model. Numerical experiments and discussion are shown in Section 4, and the fusion performance is evaluated by visual observation and quantitative evaluation. The conclusion is given in Section 5.

## 2 RELATED WORKS

### 2.A Saliency detection

Human vision perception system is highly sensitive to contrast in visual signals, such as color, intensity, and texture. Based on this observation, the saliency values of image pixels are defined by using color statistics of the input images.^24^, ^25^ The saliency value of a pixel $I_{k}$ in an image I is

(1) $$S\left(I_{k}\right)=\sum_{I_{i}\in I}D(I_{i},I_{k})$$

where $D(I_{i},I_{k})$ denotes the color distance metric, and i_s_ calculated by the absolute value of the color difference between pixels $I_{k}$ and $I_{i}$ . The saliency of a pixel is defined by using its color contrast to all other pixels in the image.

For gray input images, the color distance metric $D(I_{i},I_{k})$ is calculated by the intensity difference. When using the intensity values to rearrange the pixels of the image I , the pixels with the same intensity value $c_{l}$ are grouped together, and they have the same saliency value ${\text{S(c}}_{l})$ .

**Fig. 1:**
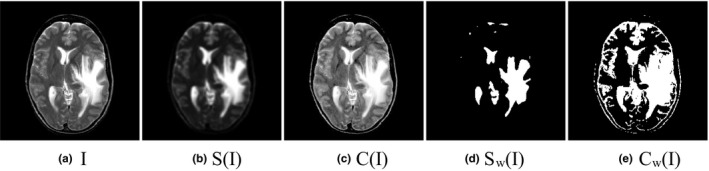
The saliency intensity feature detection, and the corresponding binary maps.

Let the intensity value of the pixel $I_{k}$ be equal to $c_{l}$ , then the Eq. (1) can be rewritten as

(2) $$S\left(I_{k}\right)=S\left(c_{l}\right)=\sum_{j=0}^{n}f_{j}D\left(c_{l},c_{j}\right)$$

where n is the number of intensities and $f_{j}$ is the probability of the pixel intensity value $c_{j}$ appearing in the image I . The saliency value ${\text{S(c}}_{l})$ is calculated by the gray difference, and the pixels with high intensity have high contrast. So, the high intensity regions can be detected globally in the saliency map S(I). In Fig. 1, we compare the saliency maps obtained by the different methods. Fig. 1(a) is a medical MR‐T2 image I , (b) is the saliency map generating by the intensity distance metric, (c) is the distance between I and its mean value, (d) and (e) are their binary map after a threshold method. By comparing (d) and (e) in Fig. 1, we can observe that there are many unexpected blocks in the map $C_{w}\left(I\right)$ for detecting the high‐intensity regions of the source MR‐T2 image.

### 2.B Structure tensor

Given a gray image I:Ω→[0,1] , for every pixel $x=(x_{1},x_{2})$ , and I(x) is the intensity of the input image I at x. The gradient ∇I is defined as follows to describe the contrast change

(3) $$\nabla I={\left[\frac{\mathrm{dI}}{dx_{1}},\frac{\mathrm{dI}}{dx_{2}}\right]}^{T}={\left[I_{x_{1}},I_{x_{2}}\right]}^{T},$$

When the distance of adjacent two pixels in the image I becomes infinitesimal, the differential dI can be represented as

(4) $$dI=\frac{\partial I}{\partial x_{1}}dx_{1}+\frac{\partial I}{\partial x_{2}}dx_{2},$$

and its square norm is

(5) $${\left|dI\right|}^{2}=\sum_{i,j=1}^{2}\left(\frac{\partial I}{\partial x_{i}}.\frac{\partial I}{\partial x_{j}}\right)dx_{i}dx_{j}=\left[dx_{1}\right.\left.,dx_{2}\right]G\left[dx_{1}\right.,{\left.dx_{2}\right]}^{T},$$

G is called as the structure tensor, and can be expressed as

(6) $$G=\left[\begin{array}{cc} {\left(I_{x_{1}}\right)}^{2} & I_{x_{1}}I_{x_{2}}\\ I_{x_{1}}I_{x_{2}} & {\left(I_{x_{2}}\right)}^{2} \end{array}\right],$$

**Fig. 2:**
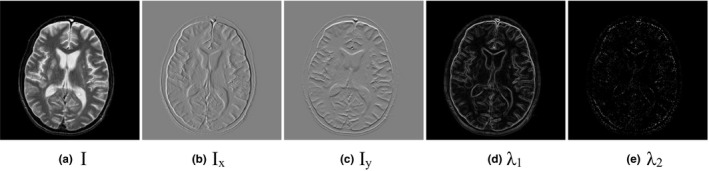
The contrast changes in different directions. (a) is the input image, (b)–(c) are the gray changes in different directions, they are x‐direction, y‐direction, θ1‐direction and θ2‐direction, respectively.

And, as a semi‐definite matrix, the structure tensor G can be decomposed as

(7) $$G=QAQ^{T}=\left(\theta_{1},\theta_{2}\right)\left(\begin{array}{cc} \lambda_{1} & 0\\ 0 & \lambda_{2} \end{array}\right){\left(\theta_{1},\theta_{2}\right)}^{T}$$

where $\lambda_{1}$ and $\lambda_{2}$ are eigenvalues, $\theta_{1}$ and $\theta_{2}$ are the corresponding eigenvectors. The eigenvalues $\lambda_{1}$ and $\lambda_{2}$ , respectively represent maximum and minimum contrast change rate of the gray image I . The gradient at pixel p can be calculated by

(8) $$ST\left(p\right)=\theta_{1}\left(p\right)\sqrt{{\left(s_{1}+s_{2}\right)}^{2}+\alpha {\left(s_{1}-s_{2}\right)}^{2}},$$

where $s_{1}=\sqrt{\lambda_{1}\left(p\right)}$ , $s_{2}=\sqrt{\lambda_{2}\left(p\right)}$ and α is a positive parameter. In Fig. 2, we calculate the gradient values in different directions. Figure 2(a) is an input image, (b)‐(e) are the gradient values of the horizontal direction, the vertical direction, the $\theta_{1}$ ‐direction and $\theta_{2}$ ‐direction, respectively. As shown in Fig. 2(d), the gradient information calculated by the structure tenor can describe the contrast changes well.

## 3 PROPOSED FUSION METHOD

In this section, we describe the implementation of the proposed medical image fusion method in details. The goal of the proposed method is to preserve the salient intensity information and clear soft tissue structures from CT and MR images. First, the contrast based method and structure tensor are used to capture the multiple intensity and structure features, and the weighted multiple features fusion is performed. Then the generated image is used as an initial input of the variational model, and the final fused image can be obtained by solving the variational model using the optimization algorithm.

### 3.A Weighted fusion based on multiple features

**Fig. 3:**
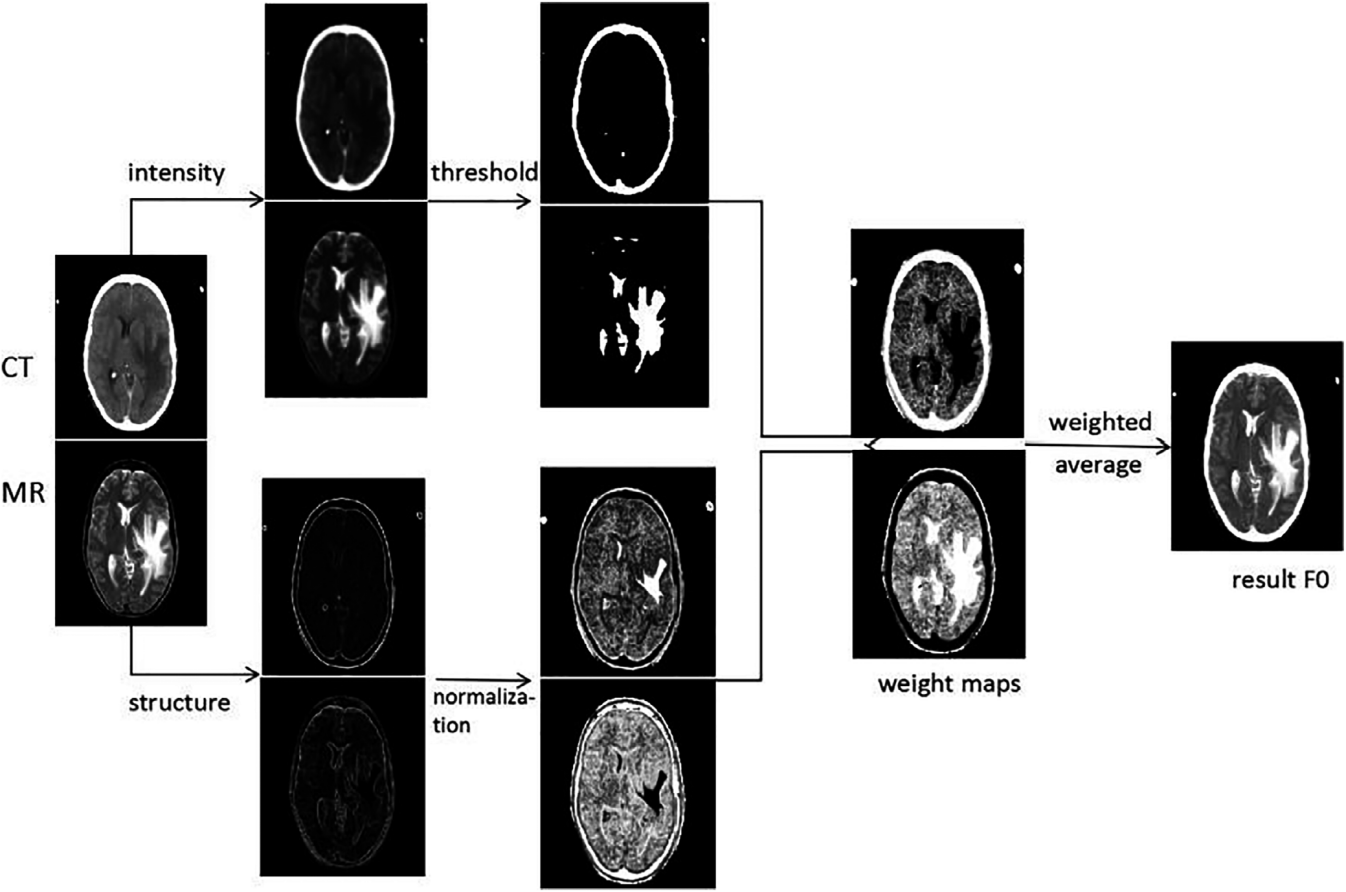
The weighted fusion process.

In image processing, the contrast feature can be used to detect the saliency intensity information, and the structure tensor is an effective tool to describe image geometry structures. In this section, the contrast and structure features are detected to generate the weight maps for weighted image fusion. Given two input images $I_{\mathrm{ct}}$ and $I_{\mathrm{mr}}$ , the weighted fusion process is shown in Fig. 3, and the main steps to get the initial fused image $F_{0}$ are described in the following.

First, we use Eq. (2) to detect the high‐intensity regions of the input images, and the saliency maps $S(I_{\mathrm{ct}})$ and $S(I_{\mathrm{mr}})$ are segmented to generate two binary weight maps $S_{w1}$ and $S_{w1}$ . Meanwhile, the structure tensor is used to calculate the structure feature maps $ST(I_{\mathrm{ct}})$ and $ST(I_{\mathrm{mr}})$ , and the two structure weight maps $ST_{w1}$ and $ST_{w1}$ can be obtained by normalization, that is,

(9) $$ST_{w1}=\frac{ST\left(I_{\mathrm{ct}}\right)}{ST\left(I_{\mathrm{ct}}\right)+ST\left(I_{\mathrm{mr}}\right)},ST_{w2}=\frac{ST\left(I_{\mathrm{mr}}\right)}{ST\left(I_{\mathrm{ct}}\right)+ST\left(I_{\mathrm{mr}}\right)}$$

The goal of the weighted fusion is to extract the high‐intensity regions from the input medical images, and preserve the structures of other regions, so we calculate the final weight maps as follows:

(10) $$w_{1}=\left\{\begin{array}{cc} 1 & if\;S_{w1}\neq 0\;and\;S_{w2}\neq 0\\ 0 & if\;S_{w1}=0\;and\;S_{w2}\neq 0\\ ST_{w1} & \mathrm{otherwise} \end{array}\right.,w_{2}=\left\{\begin{array}{cc} 1 & if\;S_{w2}\neq 0\;and\;S_{w1}=0\\ 0 & if\;S_{w2}=0\;and\;S_{w1}\neq 0\\ ST_{w2} & \mathrm{otherwise} \end{array}\right.$$

Finally, the weight maps $w_{1},w_{2}$ are used to fuse the input medical images $I_{\mathrm{ct}}$ and $I_{\mathrm{mr}}$ , and the initial fused image $F_{0}$ is generated by the weighted average method, that is,

(11) $$F_{0}=w_{1}\cdot I_{\mathrm{ct}}+w_{2}\cdot I_{\mathrm{mr}}.$$

### 3.B Variation‐based fusion

Given the image domain $\Omega \subset R^{2}$ and a pair of input images $u_{0},u_{1}$ , we propose the variational fusion method through a minimization problem:

(12) $$\underset{u}{\arg\min}E\left(u\right)=\frac{\alpha }{2}\int_{\Omega }{\left(u-u_{0}\right)}^{2}dx+\int_{\Omega }\left|\nabla u-\nabla u_{1}\right|dx.$$

Here, the final fused image and two initial input images are denoted to be $u,u_{0}$ , and $u_{1}$ , respectively. The first term is a square norm, and it is used to constrain the intensity of the fused image u to approximate the input image $u_{0}$ . The second term is a regularization term to constrain the fused image u smooth, and preserve the gradient information of $u_{1}$ at the same time.

**Fig. 4:**
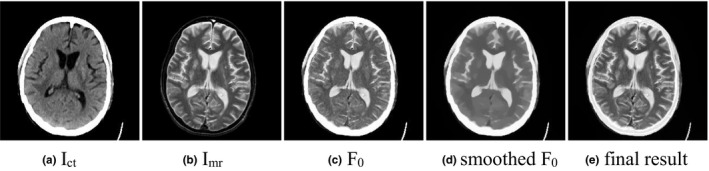
Some generated images during the fusion process.

In Section 3.A, the high‐intensity information is well preserved through the weighted‐fusion, while the tissue structures are partially missing. In Figs. 4(a) and 4(b) are two input CT and MR‐T2 images, and Fig. 4(c) are the weighted‐fusion result $F_{0}$ . Compared with the input MR image, the gray matter and white matter of the fused image $F_{0}$ are blurred, and some noise is introduced into $F_{0}$ because of different gray distributions of CT and MR images. When the TV norm is used as the regularization term, we get a smoothed result shown in Fig. 4(d). Compared with CT images, the MR images can capture clearer soft tissue texture structures. The differences between soft tissues are more distinct, such as the intensity difference between the gray matter and white matter of human brain. The contrast of soft tissues in MR images is salient. So, the structure features of the MR image $I_{\mathrm{mr}}$ are preserved in the final fused image, and the variational model (12) can be rewritten as

(13) $$\underset{u}{\arg\min}E\left(u\right)=\frac{\alpha }{2}\int_{\Omega }{\left(u-F_{0}\right)}^{2}dx+\int_{\Omega }\left|\nabla u-\nabla I_{\mathrm{mr}}\right|dx$$

The solution of problem (13) is obtained as the final fused result.

### 3.C Algorithm

The proposed variational model (13) is convex, and can be solved by the primal‐dual algorithm.^26^ The minimization problem (13) can be converted to a saddle‐point problem which is derived by duality principles:

(14) $$\min_{u}\max_{p}\frac{\alpha }{2}{∥u-F_{0}∥}_{2}^{2}+〈\nabla u-\nabla I_{\mathrm{mr}}〉-I_{{∥p∥}_{\infty }\leq 1}.$$

Let $\left(p^{t+1},u^{t+1}\right)$ be approximating solutions in the iteration process, then we have

(15) $$p^{t+1}=\underset{p}{\arg\;\max}\left\{〈\nabla u-\nabla I_{\mathrm{mr}},p〉-I_{{∥p∥}_{\infty }\leq 1}-\frac{1}{2\sigma }{∥p-p^{t}∥}_{2}^{2}\right\},$$

(16) $$u^{t+1}=\underset{u}{\arg\;\min}\left\{\frac{\alpha }{2}{∥u-F_{0}∥}_{2}^{2}+〈\nabla u-\nabla I_{\mathrm{mr}},p〉+\frac{1}{2\tau }{∥u-u^{t}∥}_{2}^{2}\right\}.$$

By applying the proximal point algorithm, the proposed model can be easily solved as follows:

**Algorithm 1:** Numerical Algorithm:

- Initialization: Choose σ>0 , τ>0 , $u^{0}$ , $p^{0}$ and set ${\tilde{u}}^{0}=0$ , ${\tilde{p}}^{0}=0$ .
- Iteration ( t≥0 ): Update $u^{t}$ , ${\tilde{u}}^{t}$ , $p^{t}$ , ${\tilde{p}}^{t}$ as follows

(17) $$\begin{array}{c} {\tilde{p}}^{t+1}=p^{t}+\sigma \left(\nabla u^{t}-\nabla I_{\mathrm{mr}}\right),\\ p^{t+1}=\frac{{\tilde{p}}^{t+1}}{\max\left\{1,{\tilde{p}}^{t+1}\right\}},\\ {\tilde{u}}^{t+1}=\frac{u^{t}+\tau \;div\;p^{t+1}+\alpha \tau \;F_{0}}{1+\alpha \tau },\\ u^{t+1}=2{\tilde{u}}^{t+1}-u^{t}. \end{array}$$

- Output: $u^{*}$ .

## 4 NUMERICAL EXPERIMENTS AND DISCUSSION

Experiments are implemented on the clinical medical CT and MR images from the whole brain atlas website (http:www.med.harvard.edu/aanlib/).^27^ Five pairs of CT and MR‐T1\MR‐T2 images of human brain are experimented to evaluate fusion performance. Each pair of images is sampled from the same slice plane and coregistered. The size of the images is 256 by 256. Besides, all the experiments are performed in MATLAB R2015b running on an Intel Core i7‐5500U CPU 2.40 GHz with 4 GB of RAM.

### 4.A Visual observation

To evaluate the fusion performance, the fusion result of the proposed method is compared with seven state‐of‐the‐art fusion methods which are TGV,^23^ GTF,^21^ MGGFF,^18^ GFF,^17^ PAPCNN,^16^ MFDF‐NSST,^14^ and LP‐SR^4^ based fusion methods, respectively.

**Fig. 5:**
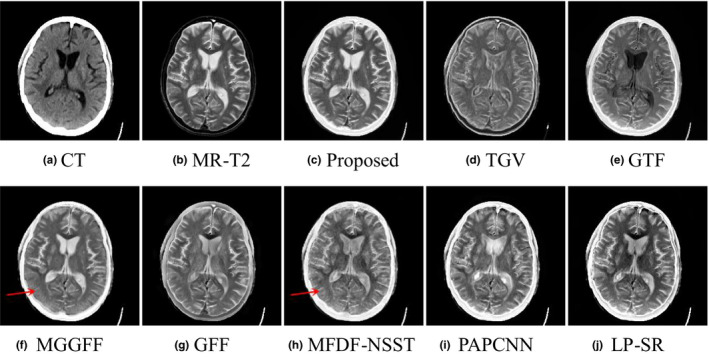
Performance comparison in the “Med‐1” experiment.

**Fig. 6:**
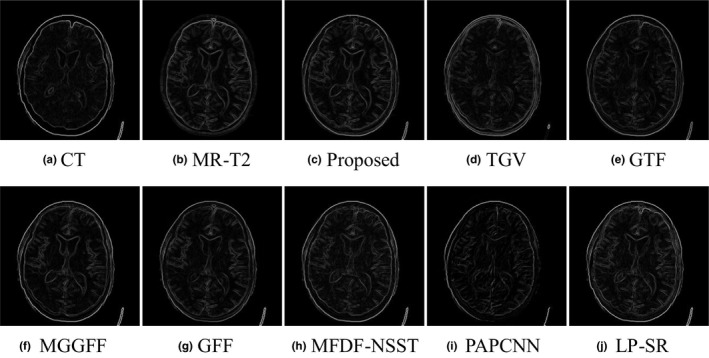
The gradient information comparison of the fused images in the “Med‐1” experiment.

As shown in Figs. 5(a) and 5(b) are a pair of input CT and MR‐T2 images, and Figs. 5(c)–5(j) are the fusion results of the proposed method, TGV, GTF, MGGFF, GFF, PA‐PCNN, MFDF‐NSST, and LP‐SR based methods, respectively. The fusion results of TGV and GTF based methods lose some intensity information, which has led to low visual quality [see Figs. 5(d) and 5(e)]. Some details are blurred in the fused images of MGGFF and MFDF‐NSST based methods, and the corresponding regions are marked with red arrows as shown in Figs. 5(f) and 5(h). In Fig. 6, the gradients of the fusion results are calculated, and the proposed method can extract smooth and clear structure information from the input images [see Fig. 6(c)].

**Fig. 7:**
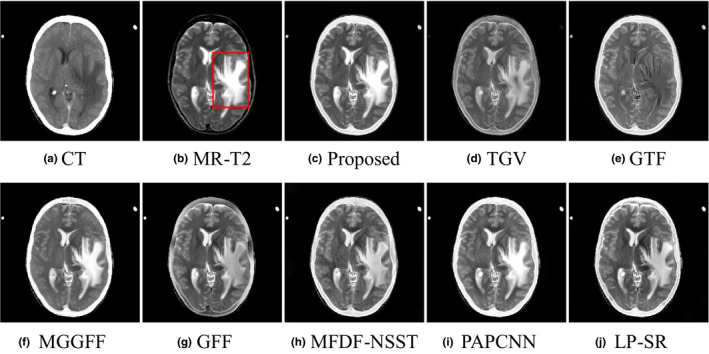
Performance comparison in the “Med‐2” experiment.

**Fig. 8:**
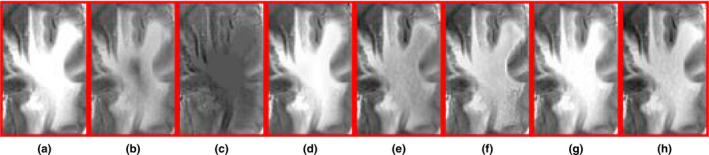
The zoomed region of fusion result images in Fig. 7.

As shown in Figs. 7(a) and 7(b) are a pair of input CT and MR‐2 images, and there exists a lesion which is reflected as one high‐signal intensity region in the MR‐T2 image ( the red rectangle). As shown in Figs. 7(c), 7(f), and 7(i), the fused images obtained by the proposed method, MGGFF and PAPCNN based methods preserve the lesion region and bone structures well without reducing the intensity contrast. The lesion regions of the fused images in Figs. 7(c)–7(j) are enlarged, and the edges of the lesion region are clearer in the fused image obtained by the proposed method [see Fig. 8(a)].

**Fig. 9:**
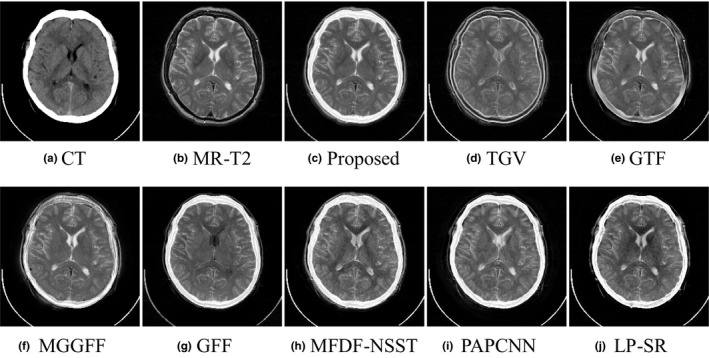
Performance comparison in the ‘Med‐3’ experiment.

**Fig. 10:**
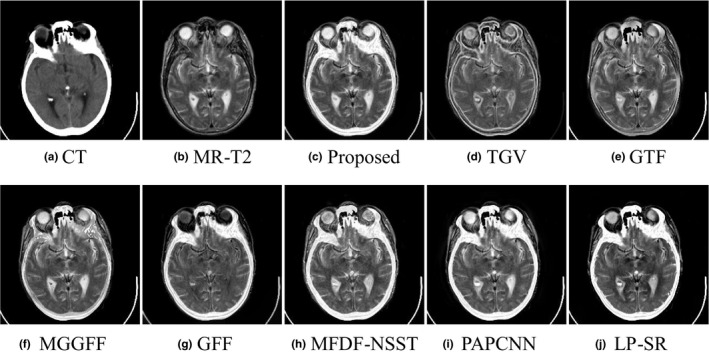
Performance comparison in the ‘Med‐4’ experiment.

In Figs. 9(a) and 9(b) are the input CT and MR‐T2 images. The proposed method can extract clear detail information from the source images. The fusion result of GFF based method preserves the soft tissues with reduced contrast. As shown in Figs. 9(h)–9(j), the detail textures are blurred in the fused images of MFDF‐NSST, PAPCNN, and LP‐SR based methods. Another set of brain CT and MR‐T2 images is experimented, and the performance comparison results are shown in Fig. 10.

**Fig. 11:**
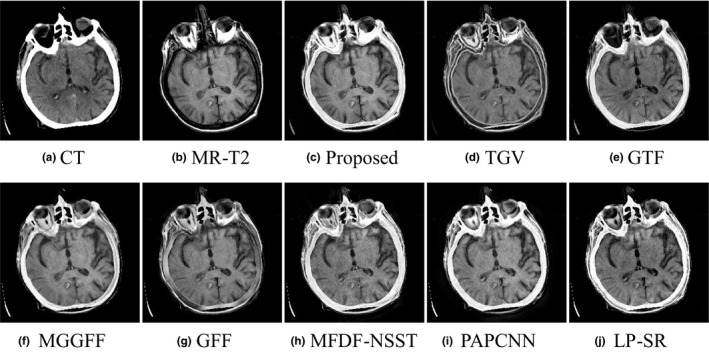
Performance comparison in the ‘Med‐5’ experiment.

Figure 11 shows the fusion results of CT and MR‐T1 images, and the proposed method can get better visual result. The experimental results in this section demonstrate advantages of the proposed method in preserving bone structures and clear soft tissues from source CT and MR images.

### 4.B Quality assessment

For objective quality assessment, we use five metrics to evaluate the fusion quality, which are spatial frequency (SF),^28^ mutual information (MI),^29^ gradient based fusion metric $Q^{AB/F}$ ,^30^ the weighted fusion quality metric $Q_{W}$ ,^31^ and visual information fidelity fusion (VIFF).^32^ The five image quality metrics are introduced as follows:

The spatial frequency (SF) is to measure the overall gray difference in an image:

(18) $$SF=\sqrt{RF^{2}+CF^{2}},$$

where RF is the row frequency and CF is the column frequency.

MI measures the degree of dependency between two variables X and Y, and it is defined as follows:

(19) $$I\left(X,Y\right)=\sum_{x,y}p_{\mathrm{XY}}\left(x,y\right)\log_{2}\frac{p_{\mathrm{XY}}\left(x,y\right)}{p_{X}\left(x\right)p_{Y}\left(y\right)},$$

where x and y are sampling variables, $p_{\mathrm{XY}}$ is the joint probability distribution for X and Y, $p_{X}$ and $p_{Y}$ are the probability distribution for X and Y, respectively. In addition, H(X)=I(X,X) is the entropy of X. For the fused image F and input images A and B, we calculate the two mutual information I(F,A) and I(F,B) , then the normalized mutual information is calculated by

(20) $$MI_{\mathrm{AB}}^{F}=2\left(\frac{I\left(F,A\right)}{H\left(F\right)+H\left(A\right)}+\frac{I\left(F,B\right)}{H\left(F\right)+H\left(B\right)}\right).$$

The metric $Q^{AB/F}$ measures the relative amount of edge information that is transferred from the input images A and B into the fused image F:

(21) $$Q^{AB/F}=\frac{\sum_{i,j}^{\mathrm{MN}}Q_{\mathrm{AF}}^{G}\left(i,j\right)W_{\mathrm{AF}}\left(i,j\right)+Q_{\mathrm{BF}}^{G}\left(i,j\right)W_{\mathrm{BF}}\left(i,j\right)}{\sum_{i,j}^{\mathrm{MN}}W_{\mathrm{AF}}\left(i,j\right)+W_{\mathrm{BF}}\left(i,j\right)},$$

where $Q_{\mathrm{AF}}^{G}\left(i,j\right)$ and $Q_{\mathrm{BF}}^{G}\left(i,j\right)$ are the edge preservation values at the pixel location (i,j) . $W_{\mathrm{AF}}\left(i,j\right)$ and $W_{\mathrm{BF}}\left(i,j\right)$ are the weights, which indicate the importance of $Q_{\mathrm{AF}}^{G}\left(i,j\right)$ and $Q_{\mathrm{BF}}^{G}\left(i,j\right)$ , respectively.

The metric $Q_{W}$ is calculated to measure how well the structural information is preserved from input images. The metric is first calculated in a local region

(22) $$Q_{W}\left(A,B,F\right)=\sum_{w\in W}c\left(w\right)\left(\lambda \left(w\right)Q_{0}\left(A,F|w\right)+\left(1-\lambda \left(w\right)\right)Q_{0}\left(B,F|w\right)\right),$$

where $Q_{0}$ is the universal image quality index, λ(w) is a local weight indicating the relative importance of image A compared to image B.

The metric VIFF is a multiresolution image fusion metric using visual information fidelity. The metric measures the effective visual information of the fusion in all blocks in each sub‐band. The metric value is calculated by integrating all the information in each sub‐band.

**Fig. 12:**
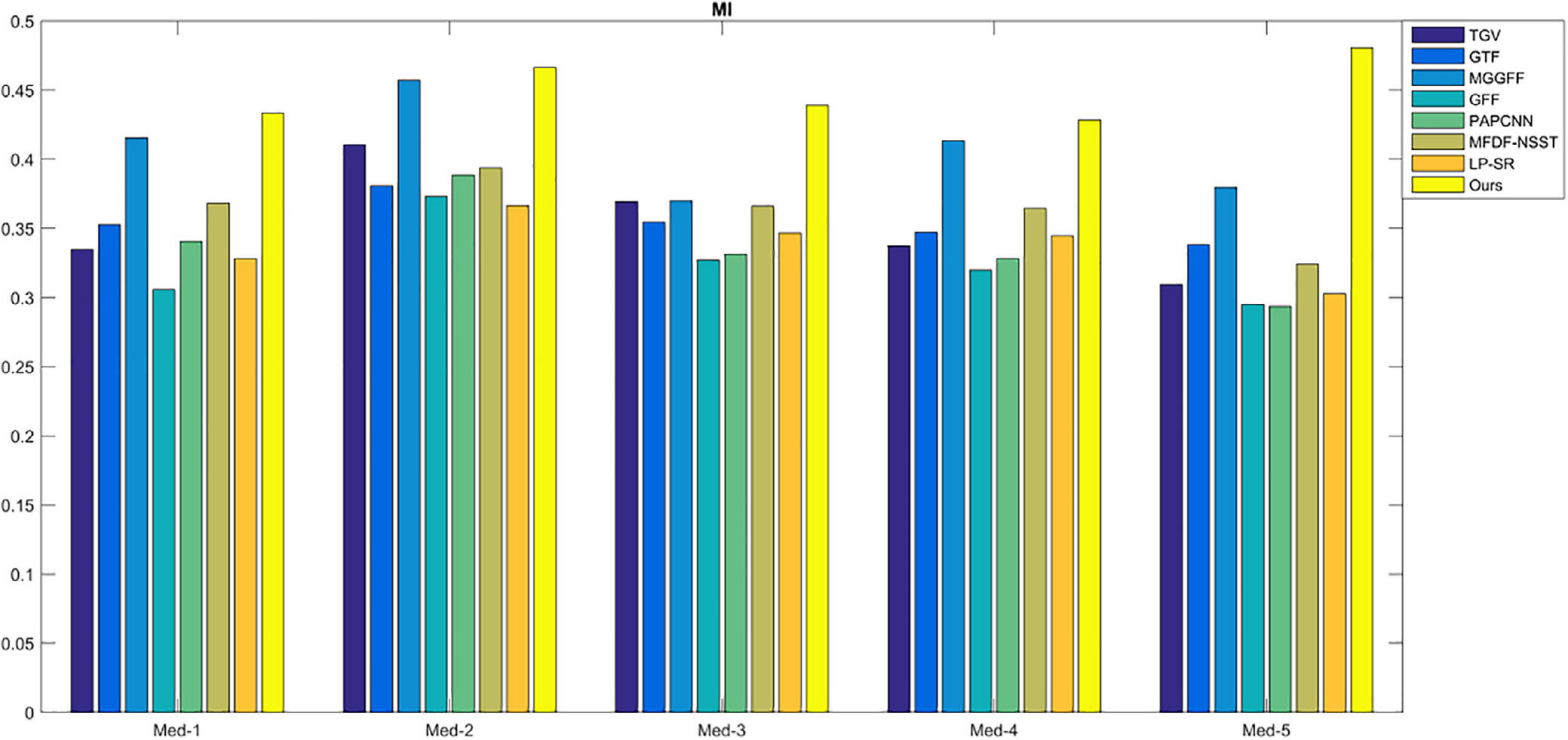
The quality assessment of MI.

**Fig. 13:**
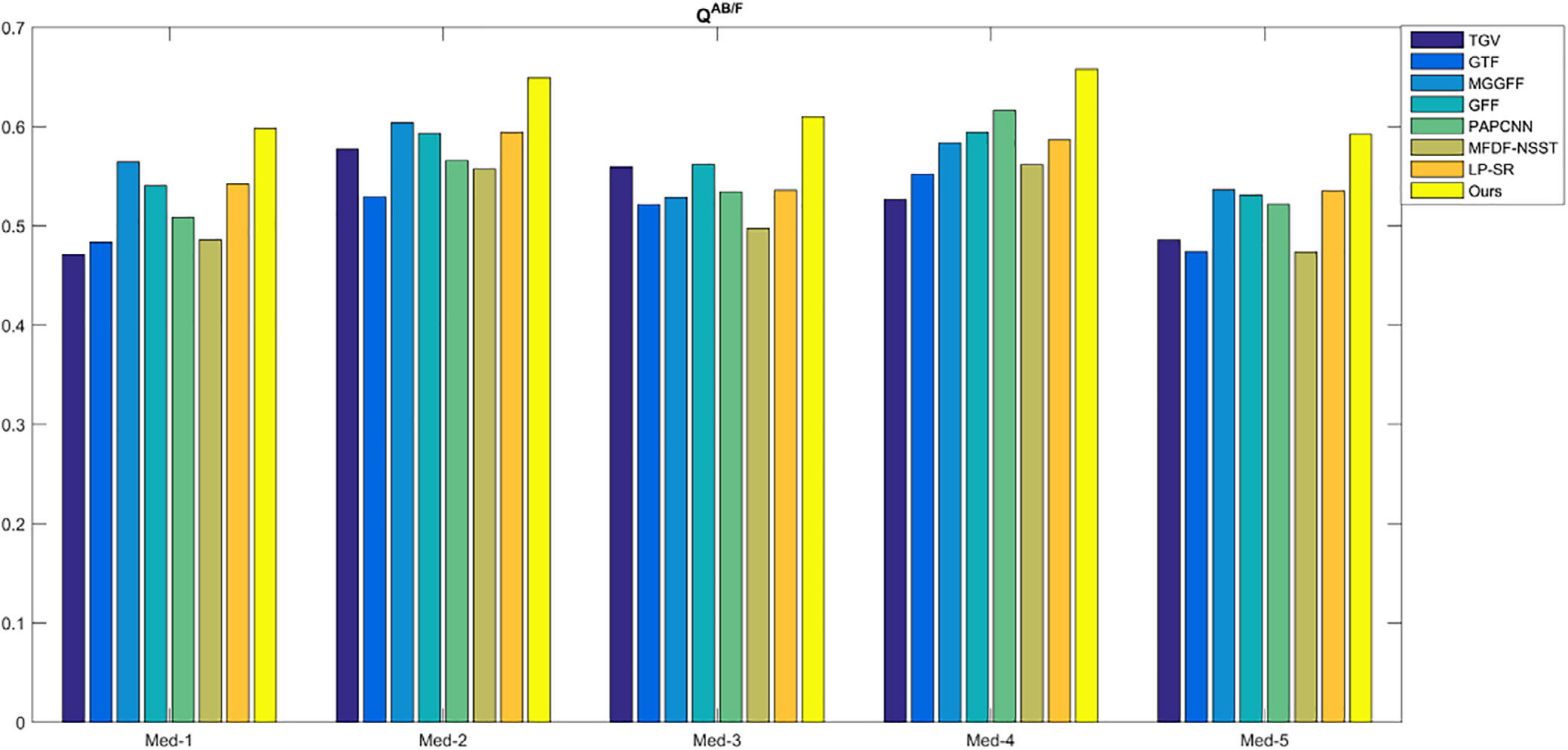
The quality assessment of $Q^{AB/F}$ .

For SF, MI, $Q^{AB/F}$ , $Q_{W}$ , and VIFF, they reflect different information including image spatial frequency, the correlation between the fused image and input images in gray level, and gradient level, the structure similarity and the visual information fidelity. For the five metrics, the values are bigger, the fusion results are better. As shown in Figs. 12 and 13, the proposed method has the best MI and $Q^{AB/F}$ values, which indicates the fused image of the proposed method preserve better gray and gradient features from source medical CT and MR images.

**Fig. 14:**
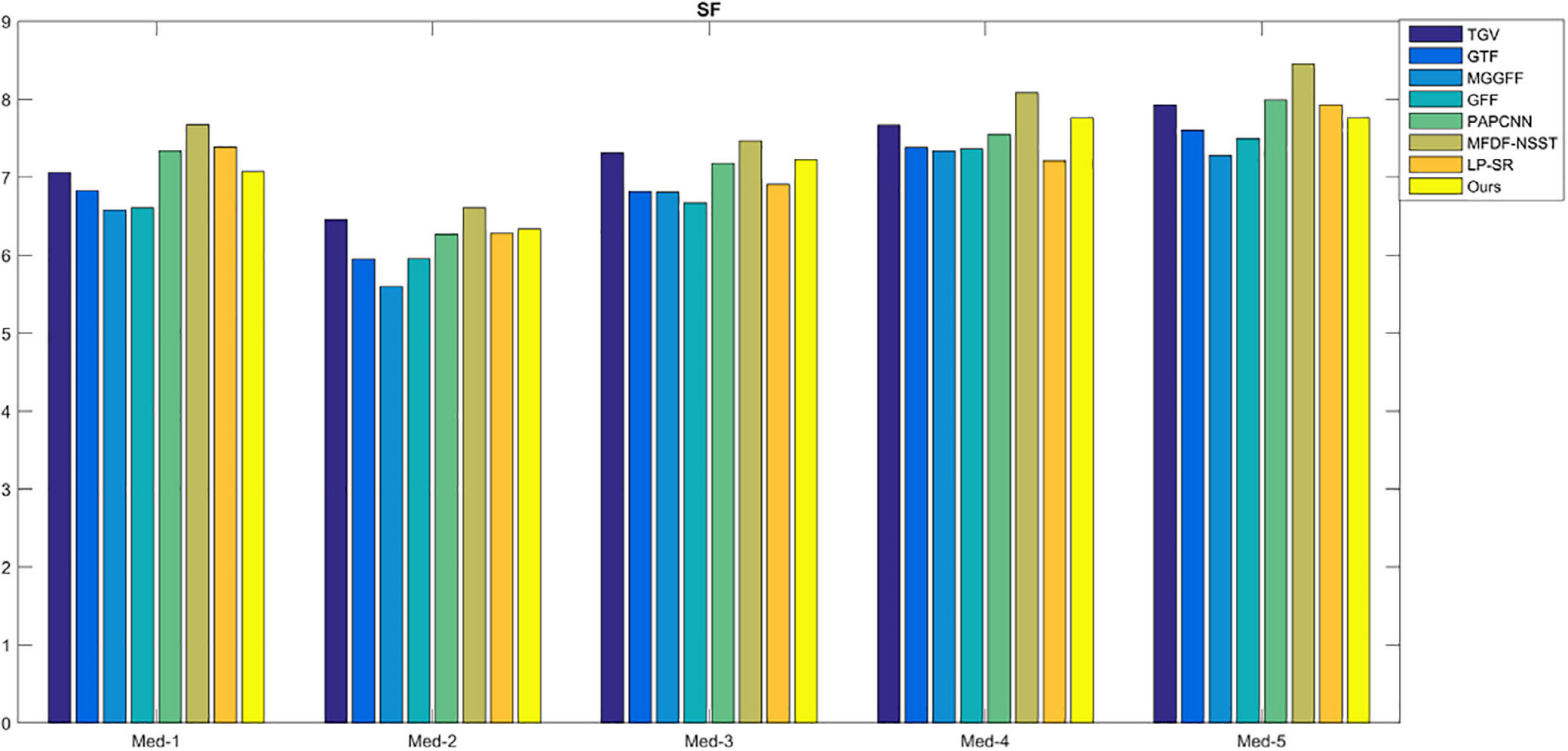
The quality assessment of SF.

**Fig. 15:**
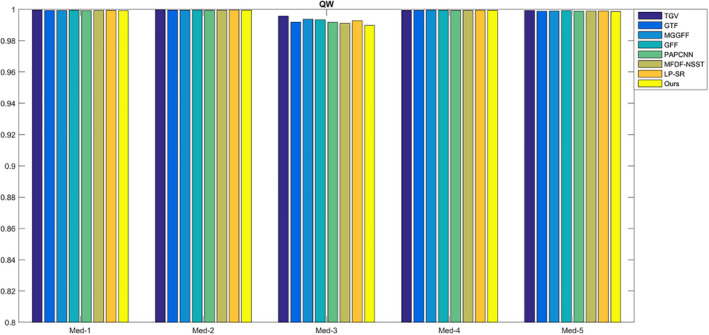
The quality assessment of $Q_{W}$ .

**Fig. 16:**
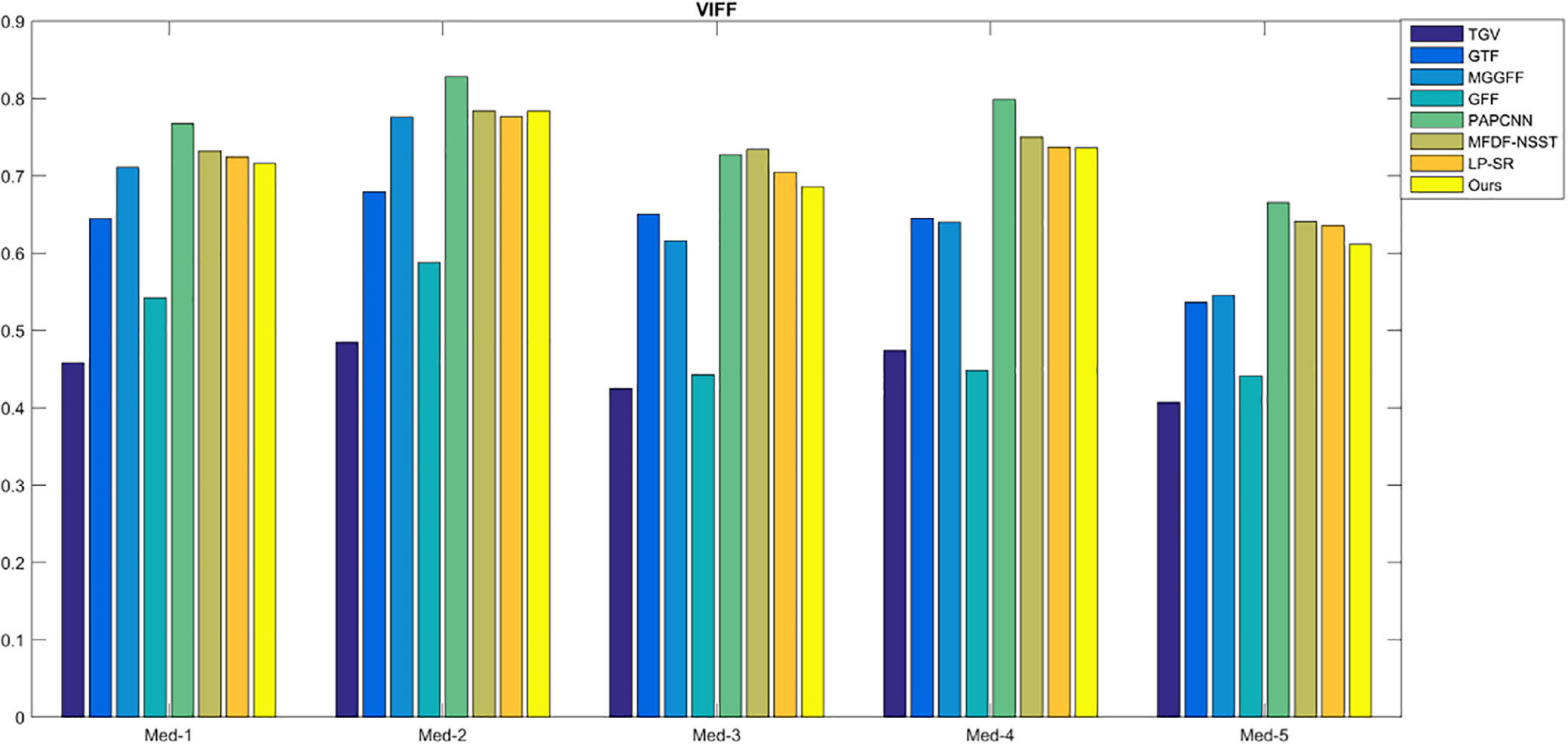
The quality assessment of VIFF.

For CT and MR images fusion, there may be artifacts in the fused image because of different gray distributions. The proposed method uses the optimization model to reduce the artifacts, so the fused results turn to be a bit smooth, and the SF values of the proposed method are lower compared with MFDF‐NSST based method (see Fig. 14). As shown in Fig. 15, the values of the metric $Q_{W}$ are too close and thus it is difficult to estimate image quality difference. The comparison of the metric VIFF is shown in Fig. 16; the proposed method has higher values compared with the seven fusion methods. In Table 1, the average running times of different methods are calculated. For LP‐SR based method, the running time is the smallest as the sparse dictionary untrained. The GFF based method costs less time because it only extracts a contrast feature for image fusion.

**Table 1:** Running time of different methods.

	TGV	GTF	MGGFF	GFF	PAPCNN	MFDF‐NSST	LP‐SR	Proposed
Times	5.3165s	1.6414s	2.7602s	0.3060s	7.0218s	35.5988s	0.1426s	5.0889s

### 4.C Discussion

For the visual observation, the proposed method can extract the salient features from source CT and MR images, such as the high‐intensity bone structures, tumor features, and soft tissue texture information, and these salient features are well preserved in the fused images, while the seven compared methods tend to generate average or blurred features in the process of fusion. For quantitative quality assessment, the proposed method has the highest MI and $Q^{AB/F}$ values. The SF and VIFF values are higher because of the smoothing effect in the fusion process. In summary, the proposed method outperforms in intensity contrast preservation and texture structure extraction. In addition, our experiments are performed on brain images, and other images will be processed in the future work.

## 5 CONCLUSION

In this paper, we propose a two‐stage fusion method for CT and MR images. The intensity and structure features are extracted from source images for initial weighted fusion, and then a variational model is applied to optimize the initial fusion result. The comparison between the proposed method and seven state‐of‐the‐art fusion methods are performed in the numerical experiments, and five comparison metrics are used for objective assessment. The experimental results and discussion show that the proposed method can well preserve the intensity and texture structure information from source images, and get a good visual effect.

## CONFLICT OF INTEREST

No conflicts of interest.
